# Electronic and optical competence of TiO_2_/BiVO_4_ nanocomposites in the photocatalytic processes

**DOI:** 10.1038/s41598-020-69032-9

**Published:** 2020-08-11

**Authors:** K. T. Drisya, M. Solís-López, J. J. Ríos-Ramírez, J. C. Durán-Álvarez, A. Rousseau, S. Velumani, R. Asomoza, A. Kassiba, A. Jantrania, H. Castaneda

**Affiliations:** 1 Department of Electrical Engineering (SEES), Centro de Investigación y de Estudios Avanzados del Instituto Politécnico Nacional (CINVESTAV-IPN), Av. Instituto Politécnico Nacional 2508, Col. San Pedro Zacatenco, Mexico, CP 07360 Mexico; 2 Institute of Molecule and Materials of Le Mans UMR-CNRS 6283, Le Mans University, 72085 Le Mans, France; 3 Instituto de Ciencias Aplicadas y Tecnología, Universidad Nacional Autónoma de México, Circuito Exterior S/N, Ciudad Universitaria, P.O. Box 70-186, CP 04510 Coyoacán, Ciudad de Mexico Mexico; 4 Department of Materials Science and Engineering, Texas A&M University, College Station, TX 77843 USA; 5 Department of Biological and Agricultural Engineering, Agrilife Extension, Texas A&M University, College Station, TX 77843 USA

**Keywords:** Energy science and technology, Materials science

## Abstract

Nanocomposites with different ratios of titanium dioxide and bismuth vanadate [TiO_2_]/[BiVO_4_] give rise to compatible electronic band structure alignment at their interfaces to ensure enhanced photoactivated charge transfer under visible light. The sol–gel method and suitable post-synthesis thermal treatments were used to synthesize different compositions with stabilized anatase phase of TiO_2_ and monoclinic scheelite polymorph BiVO_4_. Structural, electronic and optical characterizations were performed and the results were analysed as a function of the stoichiometry, in which both crystalline structures show a clear junction formation among their characteristic stacking planes. Photocatalytic and (photo) electrochemical responses of the nanocomposites were investigated and tested for the degradation of azo dyes (Acid Blue-113, AB-113) (~ 99%) under visible light radiation. The nanocomposite with a mass ratio of (1:10) shows the highest photocatalytic efficiency compared to the other compositions. HRTEM images showed marked regions in which both crystalline structures form a clear junction and their characteristic planes. However, the increase of BiVO_4_ content in the network overcomes the photocatalytic activity due to the decrease in the reduction potential of the photo-generated electrons with high recombination rates.

## Introduction

Due to rapid increase in population and the world’s rapid industrialization, natural water bodies are being heavily contaminated with industrial wastes^1,2,3^. Pollutants present in the industrial effluents are classified as toxins, carcinogens, mutagens, teratogens, and endocrine-disrupting agents^4^. Particularly, the presence of dyes in industrial effluents leads to a decreased penetration of sunlight and a reduction of the photosynthetic activities in aquatic ecosystems, as well as an increase in the biochemical oxygen demand^5^. The dyes are specified as dispersive dyes (polyester, nylon and acetate)^6^, reactive dyes (*Safira dye, HEXL*)^7^, sulphur dyes, and vat dyes (viscous)^8^. The Azo dyes are colorants that fall under all of the above-mentioned categories. They contain one or more –N=N– groups in their chemical structure, which consists of highly substituted aromatic rings^9^. To ensure water purification, several known methods are well-established: desalination, distillation, filtration, flocculation, reverse osmosis, and sedimentation^10,11^, etc. However, the chemical structure of azo dyes leads to recalcitrant characteristics, i.e., they degrade slowly under natural conditions, and they are also resistant to conventional wastewater treatment processes^6^. For this reason, Advanced Oxidation Processes (AOPs), have received a widespread attention in recent years, since they are a very convenient water treatment method that eliminates these types of contaminants^5,7^. Among AOPs, photocatalysis is considered as a green alternative to attain an efficient purification of polluted water^12,13^. Furthermore, the implementation of photoactive nanomaterials in water treatment show outstanding results, Their high specific surface area enhances both the anti-microbial activity and the electrochemical attributes, consolidating a solar assisted disinfection/decontamination process^14–16^ with an effective quality monitoring.

In this context, TiO_2_ is known as the most efficient photocatalyst compared to other photoactive metal oxides. Also, it is widely used considering its low cost and stability^17^. However, its wide band gap allows solely UV radiation harvesting, which represents only about 5% of the solar spectrum^18^. Therefore, in order to maximize the benefits obtained from visible spectrum of sun light several strategies have been explored to suitably engineer the bandgap of TiO_2_, exclusively devoted for water purification. In particular, a promising strategy is the realization of an heterogeneous photocatalyst by means of associating TiO_2_ (TO) with other semiconductors, active in the visible-light, such as : BiVO_4_, Fe_2_O_3_, GaP, or GaAs^19^.

Bismuth vanadate (BiVO_4_), is a less expensive^20^ photocatalytic material with a band gap of 2.4 eV, able to harvest visible light for photocatalysis^10,19^ as explored and reported in our former works on pure ball-milled and doped BiVO_4_^21–25^. Among the three polymorphs of BiVO_4_, the monoclinic scheelite structure (m-BiVO_4_) was synthesized by the sol–gel method and it was found to exhibit the highest photocatalytic activity under visible radiation^26^. The synthesis methods and the annealing temperatures modulate the morphology, crystalline quality, grain size and photoactivity of BiVO_4_ photocatalyst^27^. However, even by using optimized synthesis and treatment conditions, pure BiVO_4_ show a limited photocatalytic activity as for example the low degradation rate of dyes in water about 34%^28^. Since TiO_2_ is a very good adsorbent in the UV region with a compatible electronic band structure compared to that of the visible light active BiVO_4_, the realization of heterostructure assemblies may induce synergetic effects for an efficient photocatalysis. The approach was experienced to form new nanocomposites by combining two different semiconductors with compatible band structures for efficient charge transfer at the interfaces^29,30,31^. Yang et al. has identified the combination of different semiconductors to form junctions is a good strategy to have efficient charge transfer process and hence reduces the fast recombination rate in the structures and hence allows to have good efficiency^32,33^.

In this work, BiVO_4_/TiO_2_ nanocomposites with different compositions were synthesized by modified one-step sol–gel method. The morphology, the crystal structure, the electronic and optical properties were exhaustively investigated and analysed as function of the synthesis and treatment conditions. Photocatalytic investigations were carried out on model solutions with azo dyes^34–38^ such as Acid Blue 113 and the efficiency of the dye’s degradation is evaluated and discussed as function of the features of the nanocomposites. It has been observed that these novel BiVO_4_/TiO_2_ nanocomposites with good crystalline behaviour and visible light absorption are highly efficient in the photocatalytic degradation and has shown above 80 percentage of degradation within 20 min of visible irradiation with a composite dosage of 1 g/l. With an increased concentration of 5 g/l of the nanocomposite, it is observed a complete degradation of the organic azo dye Acid blue 113. This rapid activity without using any scavengers makes this composition attractive for water treatment. Also, electrochemical measurements were carried out for a consistent analysis of the electronic structure of the heterostructures for first time. Open circuit potential (OCP) measurements performed under visible light were analyzed to understand the charge transfer mechanism and the stability of the photo-generated charge carriers. The Transmission electron micrographs with the numerically simulated planes is in good agreement with the experimentally observed alignment corresponding to the formation of the heterojunction structures of the synthesized materials.

## Experimental details

### Chemicals

Bismuth nitrate penta-hydrate (Bi(NO_3_)_3_·5H_2_0) ≥ 98.0%, ammonium metavanadate (NH_4_VO_3_) ≥ 99.0%, anatase titanium dioxide nano powder (TiO_2_) < 25 nm, 99.7%, citric acid < 99.5%, HNO_3_ (68%) ammonium hydroxide (NH_4_OH) with a 28–30% NH_3_ basis, and Acid blue 113 (50%) were purchased from Sigma-Aldrich Corporation and used as it is without further purification.

### BiVO_4_ preparation

BiVO_4_ nanoparticles were prepared by the sol–gel method by using 0.01 mol Bi(NO_3_)_3_·5H_2_0 dissolved in 50 ml of HNO_3_(10%) and adding 0.02 mol of citric acid. The mixture was kept under stirring until a colourless solution was observed (solution A). In a different beaker, 0.01 mol of NH_4_VO_3_ was dissolved in 50 mL of deionized water and then heated at 80 °C with magnetic stirring until a clear lemon-yellow solution was obtained. Subsequently 0.02 mol of citric acid was added, until a brown solution was obtained (solution B). The solution A was then added dropwise to the solution B leading to a final mixture with an intense blue coloration. Followed by the pH adjustment with a NH_4_OH solution, until the mixture reached a pH of 6.5. Then the mixture was kept under constant stirring at the same temperature until the gel formation, which was afterward dried at 100 °C overnight. The obtained powder was calcinated for 2 h at 500 °C and finally ground and kept in a desiccator prior to its characterization and use in photocatalytic tests.

### Nanocomposite synthesis

**Figure 1:**
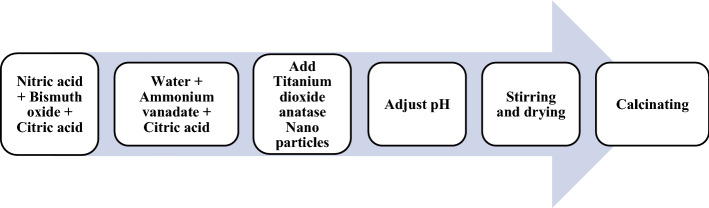
Method used to synthesize nanocomposites of BiVO_4_/TiO_2_.

Three BiVO_4_/TiO_2_ composites with different nominal molar ratios were selected from our previous experience and fixed to 1:10, 1:2.5 and 1:0.6. The different ratios were prepared by maintaining a constant concentration of bismuth vanadate in the precursor (section "BiVO_4_ preparation") and changing the amount of the added titanium dioxide. The procedure for the synthesis of the nanocomposites was conducted according to the BiVO_4_ synthesis protocol. Prior to the pH adjustment stage, the anatase mass corresponding to the ratio to be synthesized (1:10, 1:2.5 and 1:0.6) was incorporated (Fig. 1). Subsequently, the mixture was placed in an ultrasonic bath for 30 min to allow the components of the mixture to blend perfectly. Next, pH adjustment, drying, and calcination of the sample were performed as outlined in the previous section.

### Characterization techniques

The structural characterizations of the synthesized samples were performed using X-ray powder diffractometer (Bruker-D2 Phaser) at 30kv with Cu Kα radiation (λ = 1.5418 Å). The diffraction patterns were recorded as a function of 2θ ranging from 10° to 80° with a step interval of 0.02°. Using the diffraction line width (FWHM) we can estimate the size (D) of crystalline domains through Scherrer’s equation:

1 $$D = {\raise0.7ex\hbox{${K\lambda }$} \!\mathord{\left/ {\vphantom {{K\lambda } {\beta {\cos}\left( \theta \right)}}}\right.\kern-\nulldelimiterspace} \!\lower0.7ex\hbox{${\beta {\cos}\,\left( \theta \right)}$}}$$

where *D* is the crystallite size, *K* is the shape factor with a value close to unity, *λ* is the X-ray wavelength, Cu Kα = 0.154 nm, *β* is the full width half maxima (FWHM) of peak at particular plane in radians and *θ* is the Bragg angle. Raman spectral studies were performed with an aim to characterize the vibrational properties of the composites. Raman spectra were recorded between 80 and 1000 cm^−1^ on a Horiba–Jobin–Yvon spectrometer (Lab RAM HR800), with an excitation radiation from He–Ne laser (λ = 632.8 nm).

The morphological properties of the powders were investigated by means of Scanning Electron Microscopy (SEM). Powder samples were dispersed ultrasonically in methanol. A few drops of the dispersion were dried directly on carbon tape to carry out the SEM analysis (MIRA3 TESCAN; accelerating voltage 10 kV). The compositions were analysed using an Energy Dispersive X-ray Spectrometer (EDS) coupled with the SEM. Optical absorbance spectra of the samples were done with an UV–Vis spectrophotometer (Agilent Carry 5,000). The specific surface area was evaluated by the Brunauer–Emmett–Teller (BET) method on a Quantachrome Autosorb 1. Before nitrogen adsorption, the materials were dried and outgassed at room temperature for overnight. The morphological and grain analysis of the nanocomposites have been carried out using the JEOL JEM 2,100 h Transmission Electron Microscope (Jeol ltd. Tokyo, Japan).

The obtained TEM images were mapped to extract the FFT pattern over all the grain area shown. For the TEM image that show a clear overlap in between two nanocomposites, the FFT pattern over all the image was extracted. The entire area was divided into regions, depending on the bright and dark channels that show the crystalline growth. Only when a certain region showed a single result of atomic plane spacing, in the calculated FFT, that area is assigned to the characteristic crystalline plane of either BiVO_4_ or TiO_2_, depending on the plane distance value reported in their respective diffraction card. To correlate this result with a computational model two slabs were generated by means of the BIOVIA Materials Studio Package, with the crystalline structure defined by both lattice parameters and space groups from BiVO_4_ and TiO_2_. The graphical resemblance along with the unitary slab thickness, extracted from the simulated crystal, cleavage parallel to the FFT calculated planes, shows that the mapped area belongs to a single crystalline phase.

### Photoelectrochemical characterization

Thin films were deposited through a suspension of the material in Nafion-ethanol solution. An aliquot of each material was dispersed over 0.24 cm^2^ ITO substrates, previously cleaned in acetone and water. All films were dried at room temperature for 24 h. Electrochemical determinations were performed in a conventional three-electrode cell. Ag/AgCl/3 M KCl electrode was used as the reference electrode [EAg/AgCl/3 M KCl = 0.210 V/SHE]. The counter electrode was a graphite rod (99.9995% pure, Alfa Aesar) and the prepared films on ITO substrates were used as a working electrode. NaClO_4_ (0.05 M) solution was used as the supporting electrolyte. The illumination was performed using a Newport Q Housing (Model 60025) equipped with a visible light lamp. The semiconducting properties of the films were estimated from the Mott–Schottky plots. The space charge capacitance of the film was potentio-dynamically measured in the dark (v = 20 mV s^−1^ at a frequency of 100 Hz). EIS measurements were performed at open circuit potential in the dark and under illumination in a frequency window between 100 kHz and 100 MHz with AC voltage of ± 10 mV (peak to peak). For the (photo)electrochemical measurements an Autolab PGSTAT 302N potentiostat was used.

### Photocatalytic tests

The photocatalytic activity of the pure TiO_2_, BiVO_4_ and BiVO_4_/TiO_2_ nanocomposite was studied with the help of a full reflective solar simulator equipped with a 1.6 kW Xenon Arc Ozone free lamp (SCIENCETECH VHE-NL-200). In the first step, the sample with the highest photocatalytic activity was selected using Pyrex glass vessels that contained 100 ml of the AB113 solution with an initial concentration of 40 mg/l, along with the amount of the nanocomposite required to obtain a concentration of 1 g/l. Before irradiation, the sample was stirred in order to reach an adsorption–desorption equilibrium. Afterwards, the suspension was constantly stirred for two hours until the reaction lasted. About 3 ml aliquot of the dye solution was withdrawn after a specific time interval, then the samples were filtered and their absorbance was recorded at λ_max_ = 568 nm (AB113) using a UV–visible spectrophotometer (Jasco V-670). The initial pH of the dissolution was 6 and no adjustment was made to this parameter. Once the selection of the material with the highest photocatalytic activity was made, the effect of the initial dose of the photocatalyst (1, 2, 3, 4 and 5 g/l), and the initial concentration of the dye (20, 40 and 80 mg/l) in the removal process was studied. The percentage of removal was calculated by using the following equation:

2 $$\textit{Dye removal}\left( {\% } \right) = 100\left[ {{\text{C}}_{{{0}}} - {\text{C}}\left( {\text{t}} \right)} \right]/{{C}}_{{{0}}}$$

where C_0_ and C(t) represent respectively the initial dye concentration and that at time t of irradiation.

## Results and discussion

### Structural, surface morphological and optical characterizations

**Figure 2:**
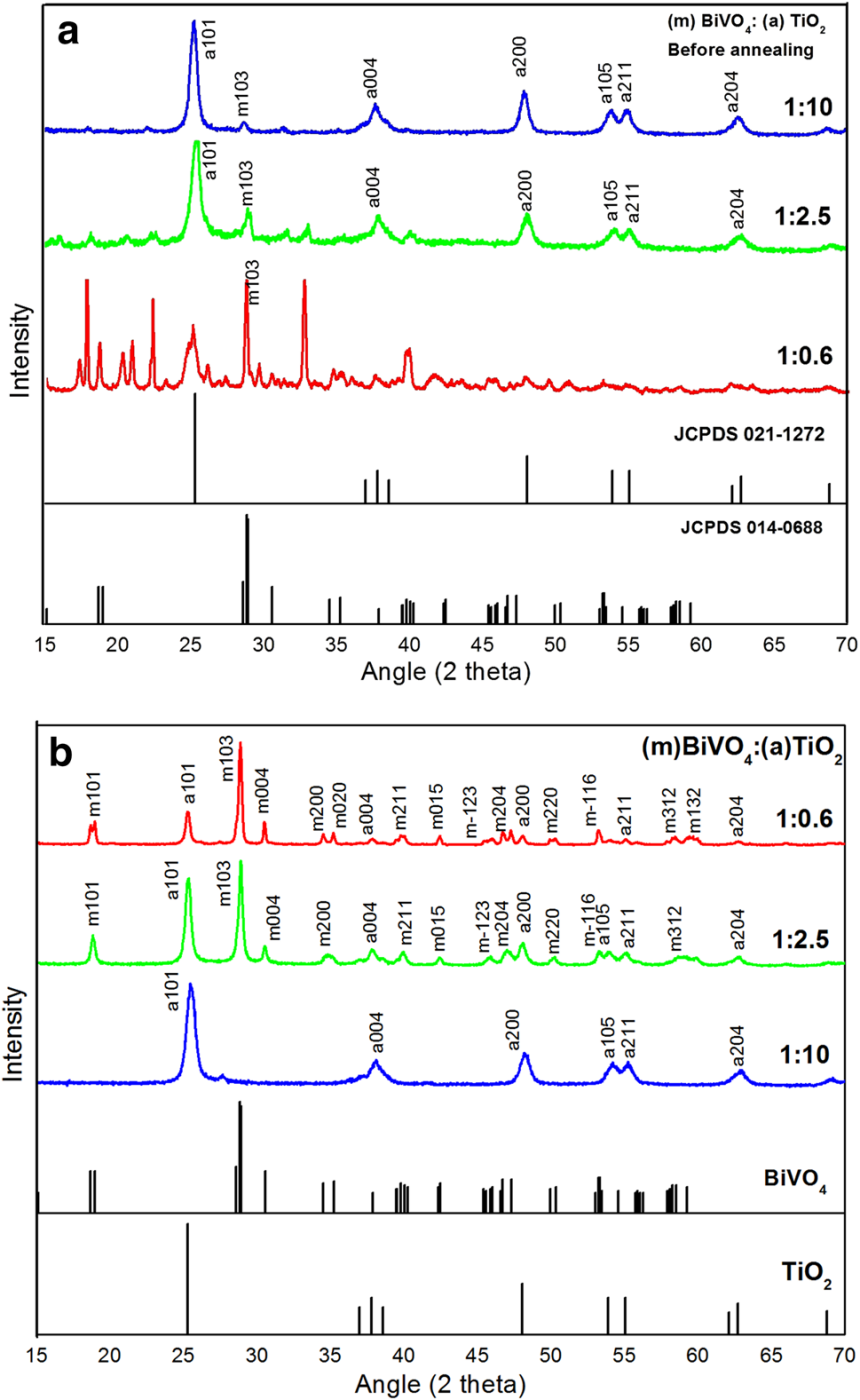
XRD patterns of the nanocomposites (**a**) before and (**b**) after calcination, compared with those of pure anatase TiO_2_ (JCPDS 00-021-1272) and sol–gel synthesized m-BiVO_4_ (JCPDS-00-075-1866). Prior to the calcination of the samples, the refinement of the patterns shows the formation of orthorhombic phase of BiVO_4_.

XRD patterns of the BiVO_4_-TiO_2_ nanocomposites before and after calcination are illustrated in Fig. 2. The refinement of XRD patterns shows that, before calcination of the samples, an orthorhombic phase of BiVO_4_ was formed (Fig. 2a). The XRD patterns (Fig. 2b) are related to the nanocomposites after calcination, compared with those of pure anatase TiO_2_ and sol–gel synthesized m-BiVO_4_. The XRD results show that, after calcination of the samples, the orthorhombic phase of BiVO_4_ was completely transformed into the monoclinic phase with reference to BiVO_4_ (JCPDS00-075-1866) and TiO_2_ (JCPDS 00-021-1272), results that match with those already reported^11^. The diffraction peaks of nanocomposites (1:10, 1:2.5, and 1:0.6) indicate the reflections related to the anatase structure, with the planes (101), (004), (200), (211), and (204) corresponding to the 2θ values: 25.28, 37.80, 48.04, 55.06 and 62.68, respectively. Concerning m-BiVO_4_, it is possible to identify the planes (101), (103), (015) and (220) of m-BiVO_4_ (JCPDS-00-75-1866), corresponding to the 2θ values 18.67, 28.6, 42.49 and 50.33. The different diffraction lines exhibit a high intensity and a narrow line width, which are indicative of good crystalline features.

Before and after calcination, all the samples show the characteristic peaks of anatase TiO_2_, which is an evidence for the stability of this structure within the samples. Prior to the calcination, the samples with the molar ratios of 1:2.5 and 1:10 show the peaks of anatase TiO_2_ as well as those of an orthorhombic phase (PDF-01-075-1418). However, before the calcination, the 1:0.6 sample exhibits both an orthorhombic phase (PDF-01-086-0392) and a bismuth vanadium oxide phase, confirming the fact that at lower temperatures there is formation of other phases beside the monoclinic one^39^. Concerning the samples that were calcinated at 500 °C, pure monoclinic BiVO_4_ was stabilized. It can be observed that, prior to the calcination, the 1:10 sample shows the very fine peaks that correspond to the orthorhombic phase of the BiVO_4_, whereas, after calcination, as shown in the Fig. 2b there is a dramatical decrease in the signal of m-BiVO_4_. This could be attributed to the difference that exists in the amount of TiO_2_ present in the nanocomposite compared to that of BiVO_4_. Moreover, based on the STEM results obtained, TiO_2_ particles tend to cover BiVO_4_ particles, which produces an attenuation of the BiVO_4_ signals present in the X-ray pattern.

Prior to the calcination process, the estimated crystallite size values establish no correlation with respect to the mass ratios of the samples (17.5, 22.08 and 21.06 nm for the 1:10, 1:2.5 and 1:0.6, respectively) this effect suggests that the nanoparticles with the orthorhombic BiVO_4_ structure, were sintered and grown during the phase transition into a monoclinic form^40^.

The crystallite size of the nanocomposites was calculated (Table 1) after calcination and it is observed that there seems to have a tendency of decrease in the crystallite size with the decreased stoichiometry of the BiVO_4_ considering its higher atomic radius. And hence the dislocation density has increased which could lead to the increase in the number of crystallites per unit area in the material.

**Table 1:** Calculated crystal properties of the nanocomposite after calcination.

Sample	2 theta	FWHM	Crystallite size (nm)	Dislocation density
0.2:0.8	25.522	0.752	10.83	8.53
0.5:0.5	25.332	0.491	16.56	3.64
0.8:0.2	25.305	0.280	29.06	1.18

**Figure 3:**
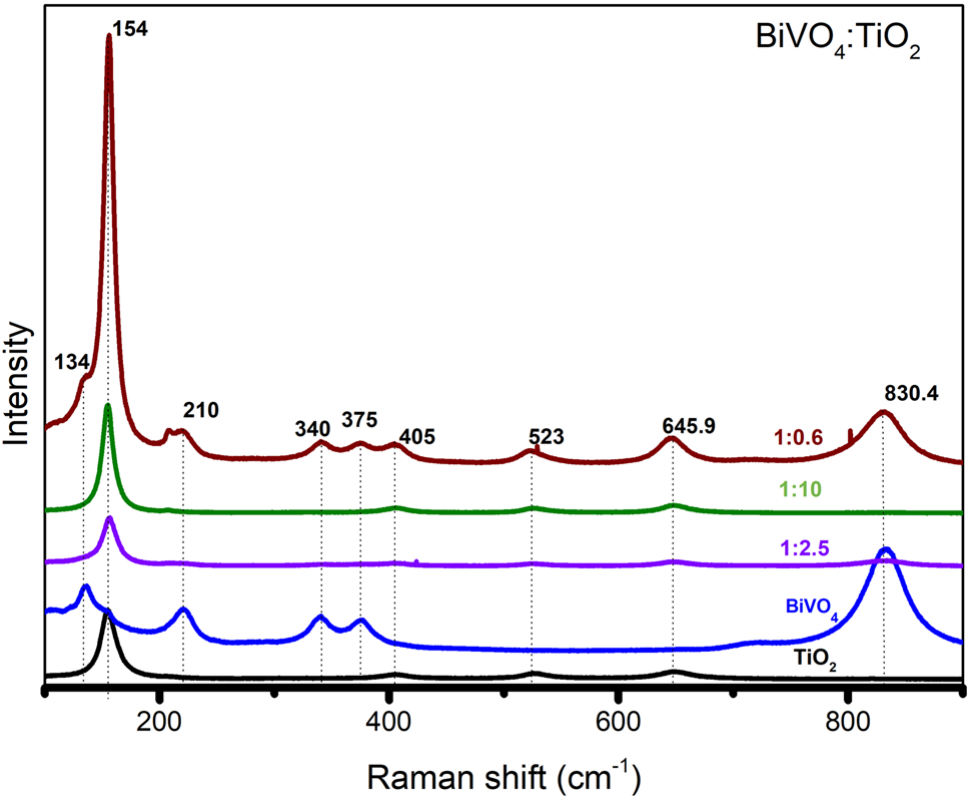
Raman spectra of TiO_2_, BiVO_4_ and its various compositions.

The Raman spectra of the nanocomposite samples with different BiVO_4_/TiO_2_ ratios are shown in Fig. 3. Among the Raman active modes, the peaks at 158 cm^−1^ and 523.3 cm^−1^ are assigned to the E_g_ (147 cm^−1^) and A1g (519 cm^−1^) modes respectively, along with a 645.9 cm^−1^(640 cm^−1^) signal, all from TiO_2_ anatase^38^. On the other hand, the bands located at 346, 375, and 846 cm^−1^ closely match with the reference by Thalluri et al. for m-BiVO_4_ (340,375 and 830.4 cm^−1^)^42^ observed the shifts for the m-BiVO_4_ at 340, 375 and 830.4 cm^−1^^21^.

The intensity of the Raman shifts at 210, 340,375, 645.9, and 830.4 cm^−1^ increases with the content of BiVO_4_. From this set of signals, both 325 and 367 cm^−1^ correspond to the bending modes of the VO_4_ groups, meanwhile, the peak at 830.4 cm^−1^ is attributed to the symmetric stretching of the V–O bond within BiVO_4_^21,25^. Finally, the Raman mode at 154.8 cm^−1^ has a remarkable inconsistency, for the 1:0.6 sample its small intensity can be attributed to a lower content of TiO_2_, whereas, a slightly intense signal from the 1:10 ratio is due to its high TiO_2_ content. The Raman mode at 154.8 cm^−1^ which corresponds to the TiO_2_ has an inconsistent change, for the 1:0.6 ratio, the smaller peak can be attributed to the lower content of TiO_2_ and that of 1:10 is little more intense where there is a high TiO_2_ content. However, in the case of 1:2.5, the Raman band shows a high intensity, that could be due to the less effect from the BiVO_4_.

**Figure 4:**
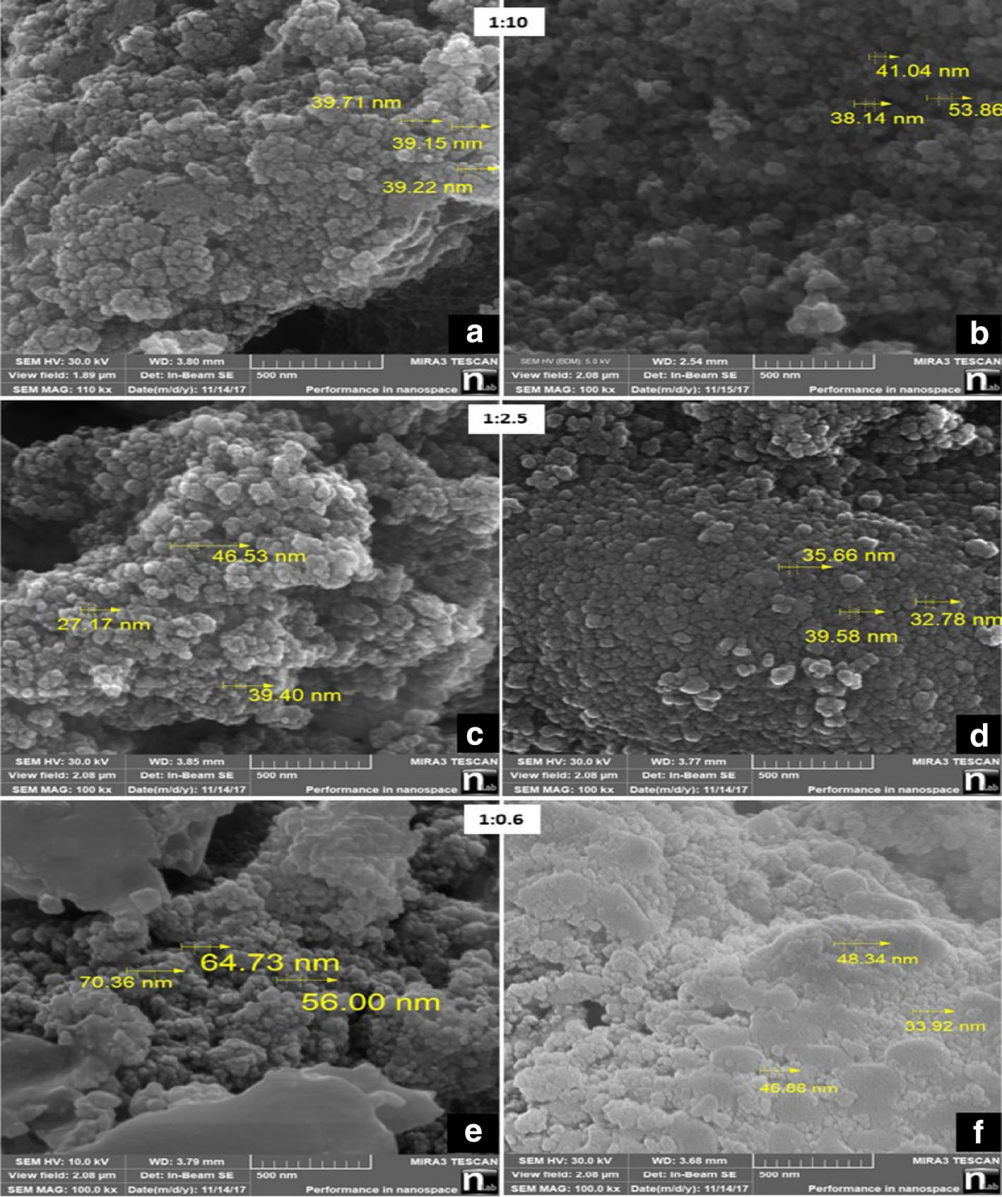
SEM images of the samples before and after calcination 1:10, 1:2.5 and 1:0.6 (**a**, **c**, **e**) before calcination (**b**, **d**, **f**) after calcination respectively.

Surface morphological analysis (Fig. 4) were carried out for BiVO_4_/TiO_2_ nanocomposites as function of the molar ratio for all samples before and after calcination which illustrates the crystal organization. Non calcinated samples possess agglomerations with barely recognizable boundaries among particles, an image that changes, with well-defined boundaries after the calcination process (Fig. 4), in agreement with a similar phenomenon observed for titanate nanotubes at T > 400^43^, associated to the coalescence of the particles during the calcination process^41^. A similar morphology reported by Rahimi et al. for a BiVO_4_/TiO_2_ nanocomposite sample calcinated at 450 °C^45^. The average particle size for the nanocomposite samples after calcination with ratios 1:10, 1:2.5, 1:0.6 were 44, 36 and 43 nm, respectively, and for the nanocomposite samples before calcination the obtained particle size was 39, 37 and 63 nm, respectively.

**Figure 5:**
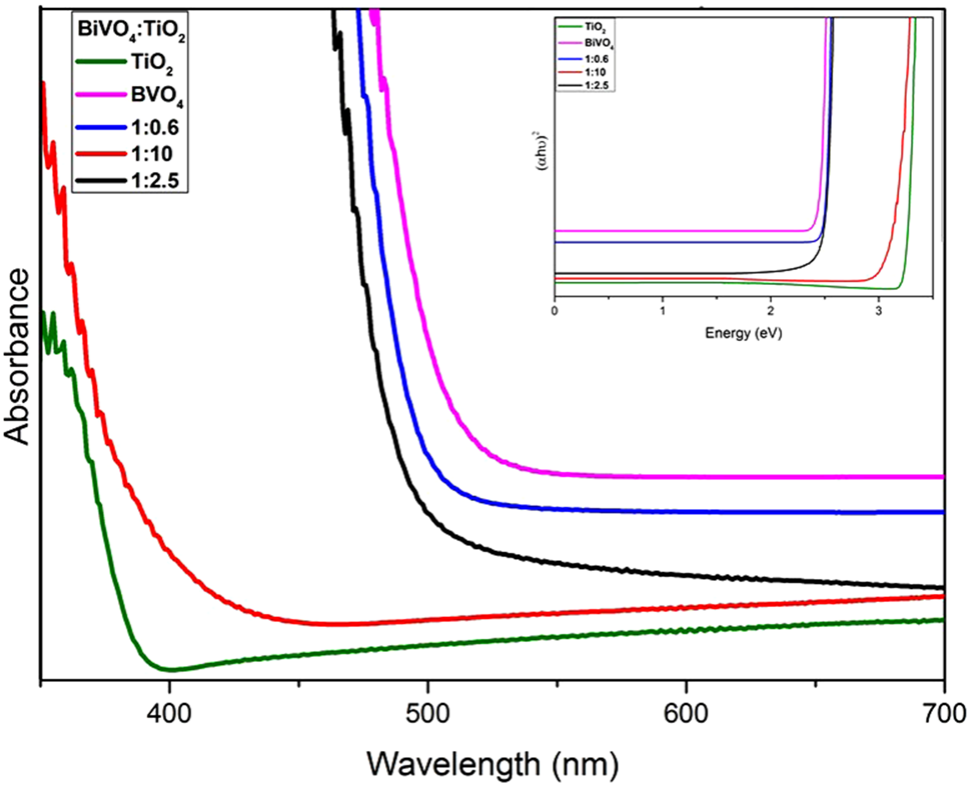
Comparison of the band gap energy of the synthesized nanocomposite samples with Anatase TiO_2_ and synthesized monoclinic BiVO_4_ nanoparticles with the Tauc plot in the inset.

Figure 5 is the plot illustrating the calculated band gap of various nanocomposites and pure BiVO_4_ and TiO_2_. The optical band gap values were calculated using the following equation^18^:

3 $$E_{g} = {\raise0.7ex\hbox{${1240}$} \!\mathord{\left/ {\vphantom {{1240} {\lambda_{g} }}}\right.\kern-\nulldelimiterspace} \!\lower0.7ex\hbox{${\lambda_{g} }$}}$$

The observed band gap value for m-BiVO_4_ was 2.34 eV, and that of pure TiO_2_ was 3.2 eV, in agreement with previous studies^24^. The absorption range of the nanocomposites lies in between that the values of pure TiO_2_ and that of m-BiVO_4_^43^. For all the nanocomposites with different ratios (1:10, 1:2.5, 1:0.6) we observed the shift in the band gap values for the nanocomposites^44^. In the case of the nanocomposite with a high TiO_2_ content, the band gap of the nanocomposite was close to the value of pure anatase, with a tendency of shifting towards the visible light range due to the presence of a higher fraction of BiVO_4_. As matter of fact, the band gap engineering of BiVO_4_/TiO_2_ shifts in the visible range as required for their efficient photocatalytic reactions. The observed band gaps for the synthesized ratios are summarized in Table 2. In the case of nanocomposites, each energy levels may have ‘n’ different energy levels, which is equal to the number of atoms interacting to each other. So in the case of nanocomposites, these bands may overlap and hence reduces the overall band gap^47,48,49^.

**Table 2:** Calculated band gaps of BiVO_4_, TiO_2_ and its various nanocomposites.

Material	Bandgap (eV)
BiVO_4_	2.34
TiO_2_	3.20
1:10	2.96
1:2.5	2.43
1:0.6	2.42

**Figure 6:**
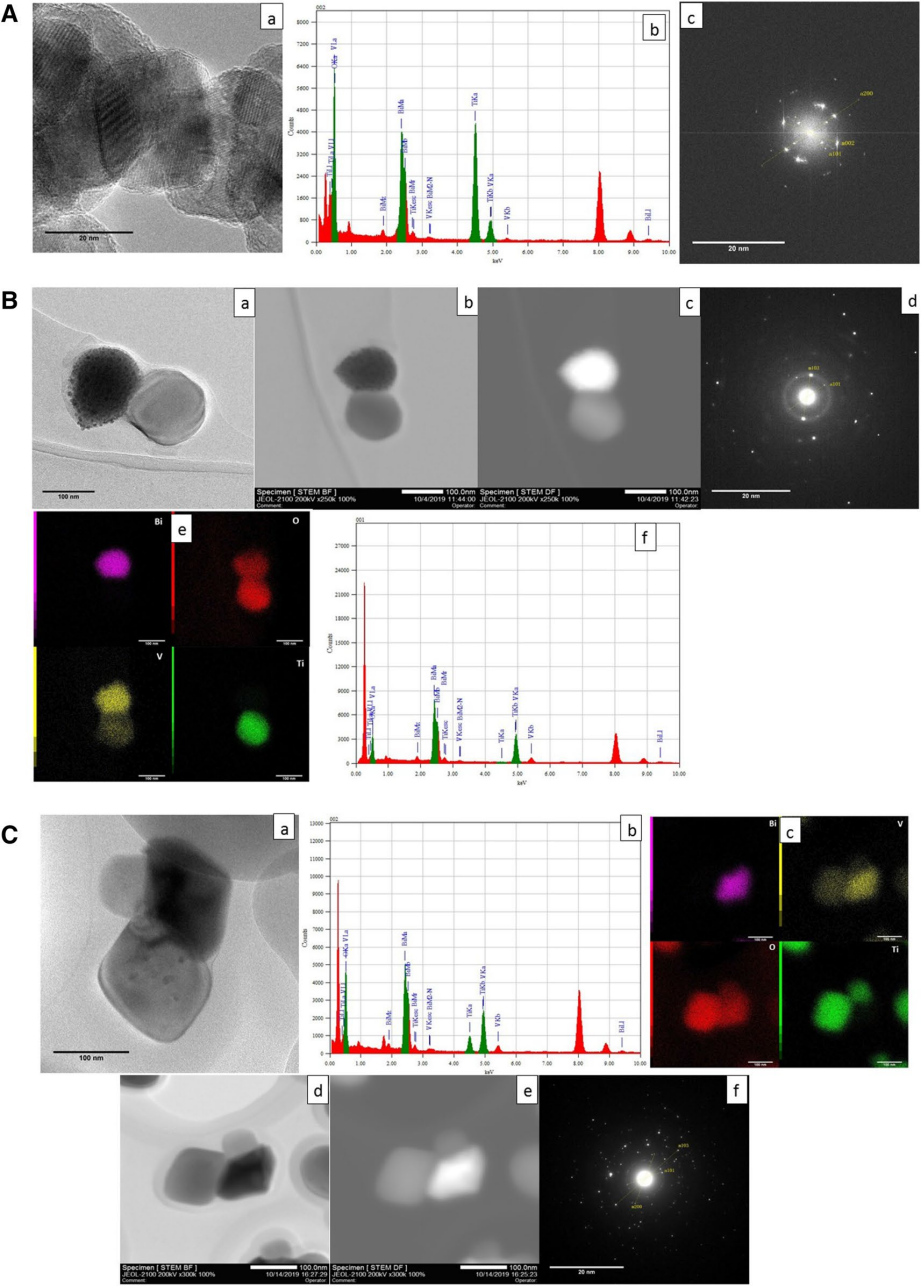
**A** Micrographs of the 1:10 nanocomposite (**a**) TEM micrograph (**b**) EDS chemical analysis (**c**) FFT diffraction pattern. **B** Micrographs of the 1:2.5 nanocomposite (**a**) TEM micrograph of isolated particles (**b**, **c**) bright and dark field analysis (**d**) FFT diffraction pattern (**e**) elemental mapping and (**f**) EDS analysis respectively. **C** Micrograph of the nanocomposite 1:0.6 (**a**) isolated particles (**b**) EDS analysis of the nanocomposite (**c**) elemental mapping of the nanocomposite, (**d**) bright-field image (**e**) dark-field image (**f**) FFT diffraction pattern.

**Figure 7:**
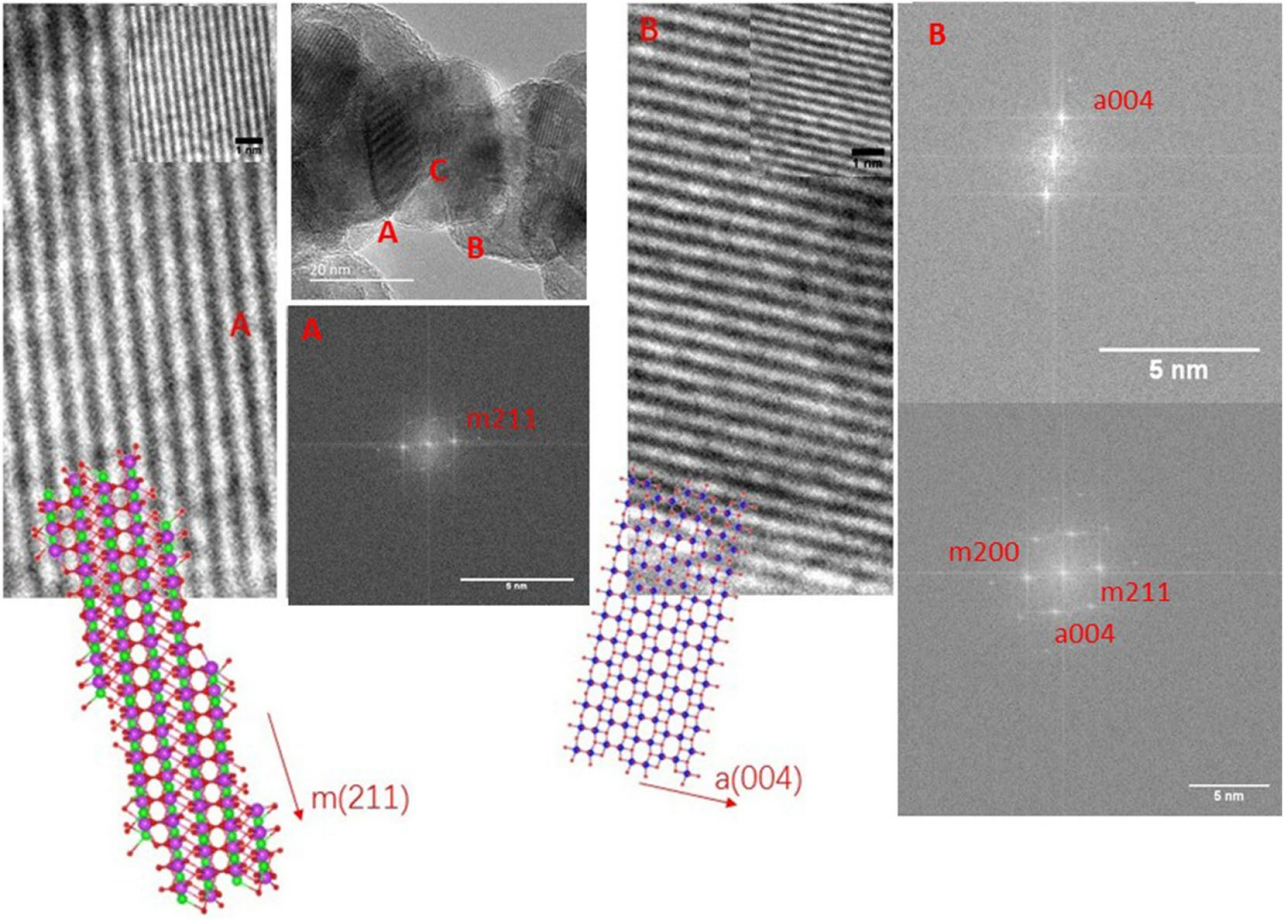
TEM micrograph, FFT planes and simulated crystalline models for the 1:10 composite. Regions (**A**), (**B**) and (**C**) corresponds to pure BiVO_4_, TiO_2_ anatase and the junction of both crystalline phases, respectively.

The transmission electron micrographs of the nanocomposites are illustrated in Figs. 6 and 7. The morphology of the particles is similar to the spherical images obtained from scanning electron microscopy (Fig. 4). The grains of composite material indicate the formation of heterojunctions associating with BiVO_4_ and TiO_2_ planes. All the reported micrographs show superimposed structures from BiVO_4_ and TiO_2_ compounds as clearly seen in EDS analysis performed on the different samples (Fig. 6B-e, C-c). In the dark and bright-field images, it is possible to see the different atomic mass contrasts (or Z contrast) involved in composites grains, even though in certain cases the contrast difference can result from the difference in the grain thickness. From Fig. 6A-a, the presence of the planes with different orientations can be seen. Figure 6A-b, the EDS analysis confirms the presence of all the elements in the composite. Figure 6A-c, FFT diffractions patterns, the planes (101), and (200) corresponding to the anatase phase of titanium dioxide and (002)^37^ corresponding to the diffractions from the monoclinic bismuth vanadate system can be seen. Figure 6B-a shows a high-resolution image of sample 1:2.5 where the crystallographic planes are identified and are in agreement with the desired monoclinic and anatase phases (Fig. 6B-d). Figure 6B-d is the FFT for the sample 1:2.5 and there observed diffraction from the planes (103) that is from the monoclinic and the (101) corresponding to the anatase phase. Figure 6B-b, B-c are the bright and dark field micrographs where the atomic mass contrast is visible and Fig. 6B-e is the mapping analysis of the sample where the presence of each element is identified by the EDS (Fig. 6B-f). Figure 6C-a is the TEM micrograph of samples 1:0.6 and Fig. 6C-b is the EDS chemical analysis of the samples in which, presence of all Bi, Ti, O, and V elements can be identified. Figure 6C-d, C-e are the bright and dark field images with the Z contrast and corresponding chemical composition can be identified from the mapping analysis Fig. 6C-c, provides the evidence of the mixed composition from BiVO_4_ and TiO_2_ with a wide area of heterojunctions. This organization is expected to favor the photo-induced charge transfer process between the coexisting structures. All the planes observed in the FFT pattern of the TEM are in good agreement with peaks observed in the X-ray diffractogram (Fig. 2). Similarly, the diffraction images for the sample 1:0.6 (Fig. 6C-f), we observed significant diffractions from the monoclinic (103) and anatase (101). The plane (101) from the anatase corresponding to the angle 25.28° is observed in all the samples which are the significant planes. And the significant one for the monoclinic is the (103) at an angle of 28.6 ^º^ and in the stoichiometry 1:10, the significant (103) plane is not visible in the diffraction image of the sample, and the same character is observed from the XRD of the sample; which attributes to the low composition of the Bismuth vanadate. The planes were identified using the standard JCPDS files used for the XRD analysis (BiVO_4_(monoclinic); JCPDS-00-075-1866 and TiO_2_ (anatase); JCPDS-00-021-1272)^11^. In all cases, the EDS and mapping (each element is identified inside the image) confirm the presence of both phases. And the synthesized materials have shown good crystallinity. The grain size from the TEM micrographs is found to be 94.82, 75, and 31.77 nms corresponding to the 1:0.6, 1:2.5, and 1:10 samples respectively. The observed large difference between the crystallite size obtained from the Scherrer calculations, which can be due to the formation of single large particles from a group of crystallites, and similar tendency can be seen from the large particles depicted by the SEM micrographs.

The clear distribution of the atomic planes, shown in the TEM image of the 1:10 composite, allows the mapping of the crystalline phases in each grain. In Fig. 7 there are three highlighted spatial regions with both pristine monoclinic BiVO_4_ and anatase TiO_2_, in region A and B respectively. For the former case, the simulated FFT pattern shows a single plane spacing, associated to (221) of m-BiVO_4_ equal to 2.26 Å, as reported for bismuth vanadate composites^50^. Meanwhile, the associated (004) plane distance is found over the pristine TiO_2_, equal to 2.29 Å, as reported in anatase based nanocomposites^51^.

The simulated crystalline structures generated by the BIOVIA Materials Studio Package, show the monoclinic clinovisvanate (space group number 88; I2b) with a cleavage over the (211) plane, from the conventional unit cell containing four Bi, four V and eight O atoms . The stacking of the (211) planes grows along a perpendicular direction to the plane defined by the vectors U [1 – 2 1] and V [0 1 − 1], with a unitary thickness equal to 2.261 Å which is in excellent agreement with the graphically determined distance. On the other hand, the anatase phase of TiO_2_ (space group number 141; I41amd) shows a stacking of (001) planes, parallel to the c lattice parameter of anatase, with an equivalent thickness equal to 2.372 Å, in agreement with the value determined by the FFT calculation. As a graphical aid both slab models are superimposed to their respective pristine regions of the TEM image. The slab model of the m(211) planes show empty channels surrounded by vanadium and bismuth atoms that match the brightest signals from region A, meanwhile, the lighter areas in the TEM image from region B could be assigned to the rings of Ti atoms along [100] and [010] directions. Such agreements support the association of the atomic plane distance to the crystalline phase at the pristine regions. However, in the portion indicated as C region the calculated plane distance in the FFT patterns evidence the junction formation since the characteristic planes from both crystalline phases appear together, as it was reported in bimetallic layered double hydroxides with MoS_2_ in a stacking array^52^ as well as in core–shell nanoparticles composed by PdO/ZnO^53^, where the presence of the interplane distances from the constituents in TEM images reveal the formation of effective coverages and an interface.

### Photocatalytic activity measurements using AB113

**Figure 8:**
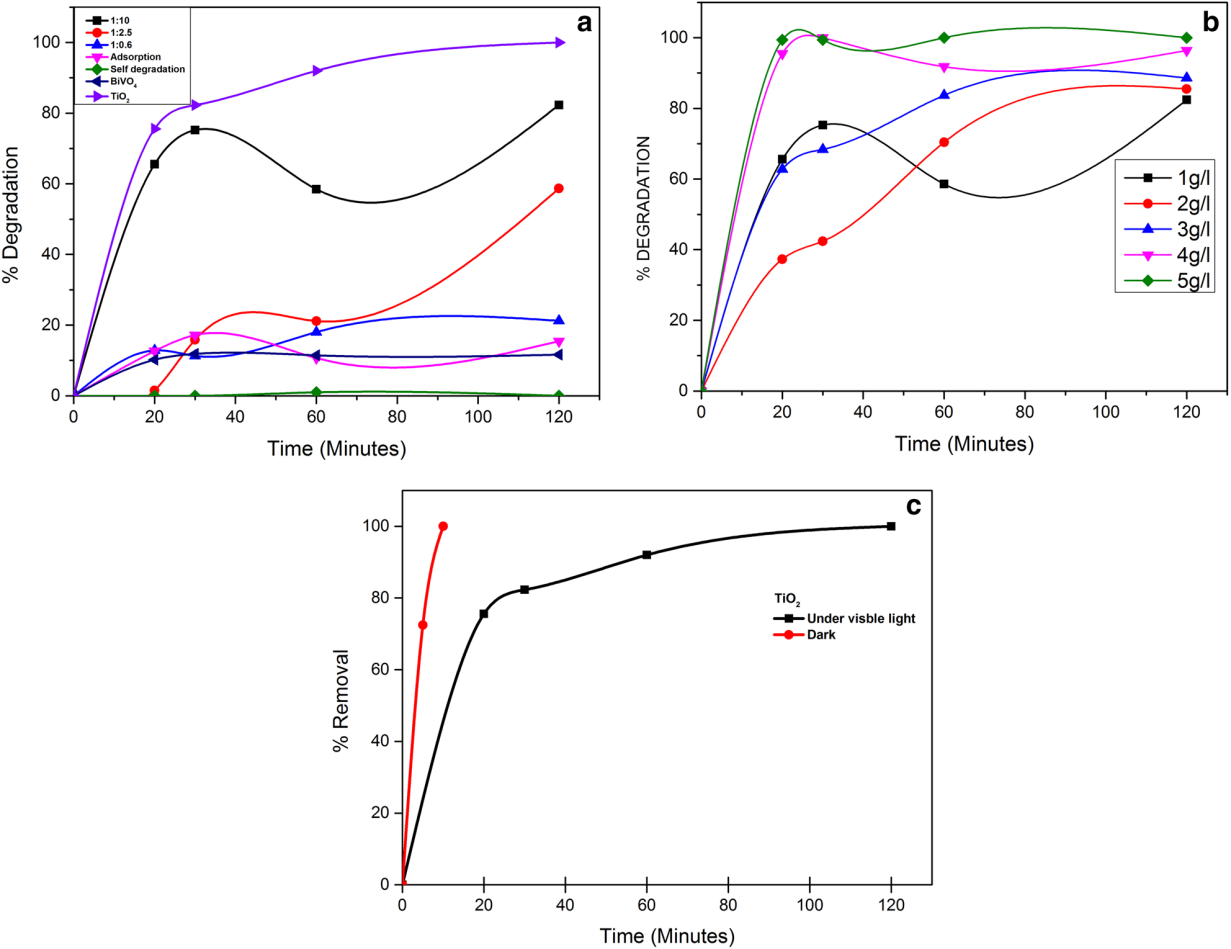
Photocatalytic degradation of AB113 (**a**) Adsorption and self-degradation plots along with the comparison of photocatalytic activity of TiO_2_, BiVO_4_ and the three nanocomposite ratios [AB113]_0_ = 40 mg/l, [nanocomposite, TiO_2_, BiVO_4_] = 1 g/l pH_0_ = 6. (**b**) [AB113]_0_ = 40 mg/l, [nanocomposite] = 1–5 g/l pH_0_ = 6 (**c**) Comparison of the activity of TiO_2_ (Both adsorption and photocatalysis).

The photocatalytic studies were conducted using the three ratios of the TiO_2_, BiVO_4_ and BiVO_4_/TiO_2_ nanocomposites (1:10, 1:2.5 and 1:0.6). Initially, we kept the dye concentration at 40 mg/l and the nanocomposite concentration at 1 g/l with the pH fixed at 6 throughout the experiment. In these conditions, the 1:10 sample demonstrated the highest rate removal of AB113 (82%) after two hours of irradiation. The sol–gel synthesized BiVO_4_ and the other molar ratios exhibited poor activity. Similar results were reported by Longo et al.^54^, who synthesized the BiVO_4_ /TiO_2_ nanocomposites using the hydrothermal method. They observed that the activity of the substance was directly proportional to the total amount of the used TiO_2_. This behaviour was attributed to the low activity of BiVO_4_ derived from its small specific surface area, 52, 56 and 44 m^2^/g for nanocomposites ratios 1:10, 1:2.5 and 1:0.6 respectively. This morphology influences the density of photogenerated charge carriers^46^ due to the recombination process. The ratio 1:10 was selected to conduct the dye removal by photocatalytic reactions since this compound showed the best results with the lowest concentration of the nanocomposite material. According to Fig. 8a, as the concentration of the nanocomposite increases, the removal rate increases as well, so when a concentration of 5 g/l is reached the concentration of AB113 is below the detection limit. The increase in the total amount of the catalyst increases the number of active sites on the nanocomposite surface which, in turn, increases the generation of ^·^OH radicals and hence the AB113 removal. This behavior was observed by other authors as well^5^.

The observed adsorption was of very less compared to the photocatalytic degradation and also there is no self degradation of the dye (Fig. 8a). Therefore, AB113 dyes removal can be attributed exclusively to the photocatalytic process using the nanocomposites compared to the performance of TiO_2_ commercial samples in photocatalytic process. Comparing with the performance commercial samples of TiO_2_ in photocatalytic process, the results show that there is a removal of 58.5 to a 76.4% which is attributed to the adsorption by the TiO_2_ particles.

Concerning the effects of the initial concentration of AB113, the investigations show that, increasing the dye concentration from 20 to 40 mg/l lead to a higher percentage of dye removal. However, when the concentration was doubled (80 mg/l), we observed a decrease in the photocataltic efficiency. The removal depends on both the dye concentration and the nanocomposite concentration. With a dye concentration of 40 mg/l, and a nanocomposite concentration of 5 g/l, we observed the maximum percentage of dye removal, 98.75%, after 10 min of visible light irradiation Fig. 8b. With a higher dye concentration, the active sites required for the ^·^OH production prove to be insufficient, i.e., it becomes necessary to increase the photocatalyst dose.

The reaction constant was determined from the corresponding kinetic plot from the reaction which follows pseudo-first order kinetics^41^.

$$ln\frac{C}{C0} ={k}_{obs}$$

where C_0_ and C(t) are the initial and final concentrations of the AB113 dye molecules for a reaction time *t*. None of the sol–gel prepared nanocomposites surpasses the activity obtained with the TiO_2_ under the used conditions. From the rate constants (k_obs_), it can be inferred that the order for the photodegradation rate is: 1:100 > 1:2.5 ≈ 1:0.6. And the degradation observed by the TiO_2_ is completely due to the adsorption process (Fig. 8c). The nanocomposite with a low relative amount of BiVO_4_ appear as more interesting candidate to photocatalytic reactions under visible light, similar results were reported by Longo et al*.*^54^. In order for BiVO_4_ to act as a TiO_2_ sensitizer, Longo et al. suggested that a good contact between both phases is necessary to ensure high interface area required for efficient charge transfer between both components.

The absorbance peak around 568 nm that represents the chromophore group present in the AB113 azo dye. Hence the quantification of the degradation percentage can be done by measuring this peak. It is reported that the hydroxyl ions are identified with good effect over the organic contaminants^55^. Previous reports says that the discoloration is happening due to the formation of 1-naphthalenol and dibutyl phythalate as bye products during the dye degradation. I-naphthalenol is produced by the direct attack of the hydroxyl radicals on the azo group; whereas the dibutyl phythalate is formed by the effective attack of the hydroxyl radicals over the aromatic rings of the naphthalene. Both the aromatic by-products did not absorb along the visible spectrum and this can back the fact that the dye molecule has degraded and decolorized during the photocatalytic reaction^56^.

### Electrochemical properties of the material

**Figure 9:**
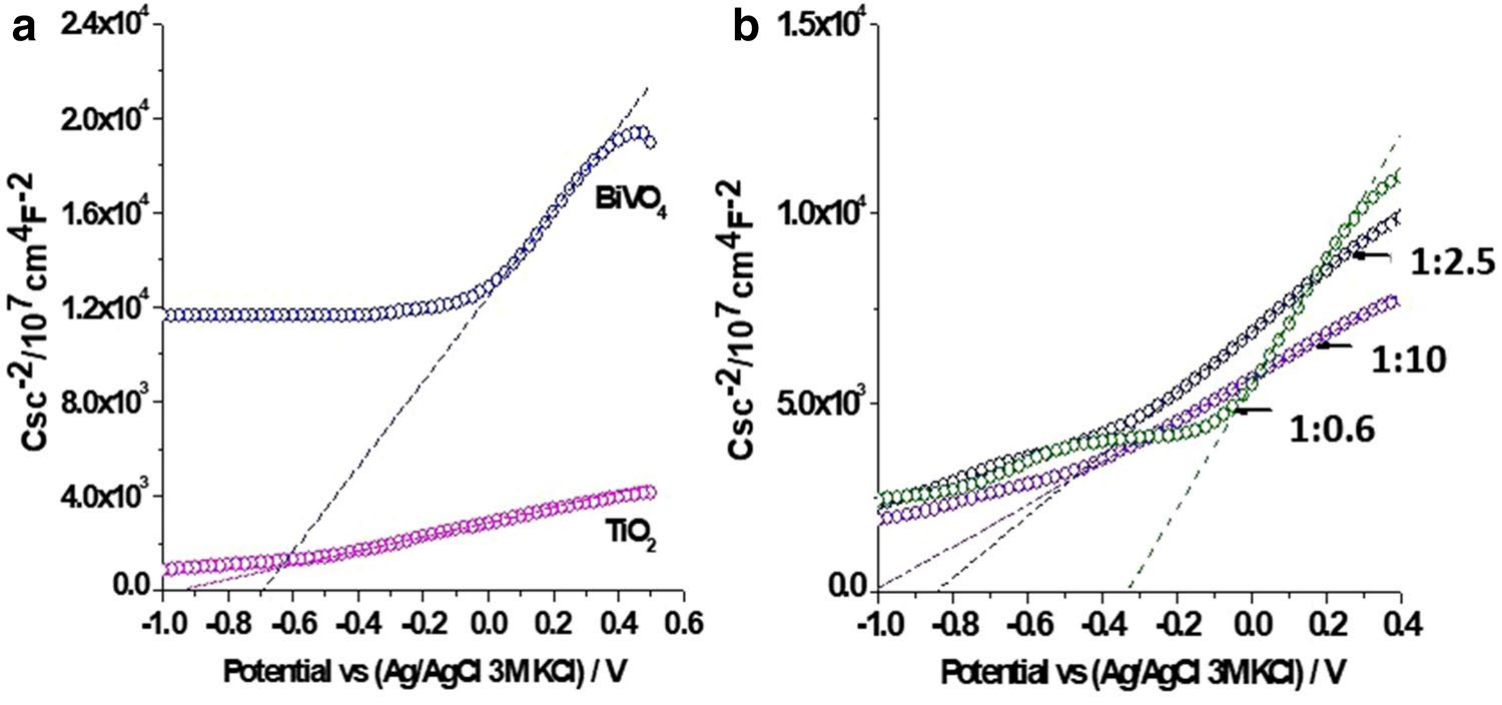
Mott–Schottky curves of (**a**) BiVO_4_ and TiO_2_ and (**b**) BiVO_4_/TiO_2_ nanocomposites (1:10, 1:2.5 and 1:0.6 molar ratio BiVO_4_:TiO_2_). The C_sc_ were obtained at 100 Hz in 0.05 M NaClO_4_ aqueous solution.

The interaction between BiVO_4_ and TiO_2_ can change the properties of BiVO_4_/TiO_2_ nanocomposites and their charge transfer process. Thus, to obtain more detail about the interface of the materials, the electrochemical impedance spectroscopy is an excellent tool. The semiconducting properties of the films were based on electrochemical impedance spectroscopy and the flat band potential appearing as the *x* intercept of the linear portion of a Mott–Schottky curves (Table 3), the response of the materials correspond to *n*-type semiconductors (Fig. 9). The inter orbital overlap or the electronic connection between the two systems are defined and explained with the help of Mott-Schottky curves, The light irradiation can generate the electron hole pairs and depending on the density, a positive and negative charged region is created forming an interface which is supposed to be due to the Schottky effect^57^. The Mott–Schottky curve is plotted (Fig. 9) with capacitance versus applied potential^58^. Intercepts of these curves provide the flat band potential. And slope of the curves give the donor or acceptor intensity depending on the type of the semiconductor (p or n-type) since the slope will be positive for the n-type semiconductors and negative for the p-type semiconductors^47^. Decrease in the slope indicates the increase in the donor density. Shift of the intercept towards the positive (Fig. 9) indicates an increase in the flatband potential^48^. The flat band potential is known as the applied voltage when there is no band bending or depletion of the charge^49^.

**Table 3:** Flat band potential of synthetized materials.

Materials	E_fb_ vs (Ag/AgCl/3 M KCl) (V)
TiO_2_	− 0.95
1:10	− 1.01
1:2.5	− 0.84
1:0.6	− 0.33
BiVO_4_	− 0.69

**Figure 10:**
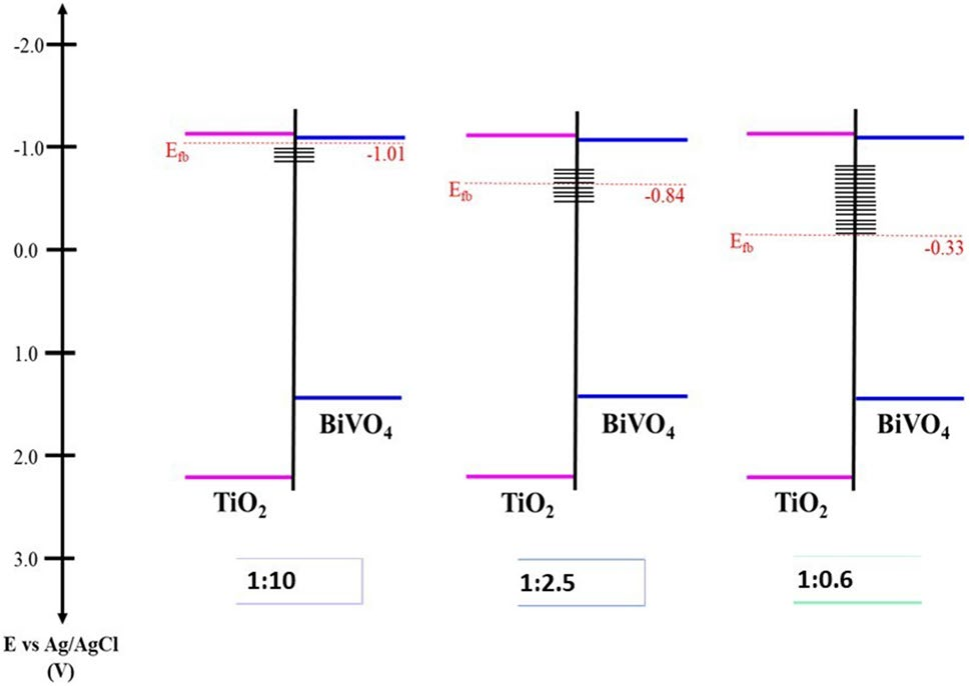
Energy diagram proposed for BiVO_4_/TiO_2_ nanocomposites (1:10, 1:2.5 and 1:0.6 molar ratio BiVO_4_:TiO_2_).

The value of E_fb_ for TiO_2_ was − 0.95 V, this value is more negative than the one reported in other work^59^. However, it can be associated with the defects in the material influenced by the synthesis techniques. On the other hand, the E_fb_ for BiVO_4_ was − 0.69 V, this value is close to a previous report^60^. For BiVO_4_/TiO_2_ nanocomposite, two different trends has been observed. First, in the nanocomposite with the lowest BiVO_4_ content (1:10), E_fb_ was displaced toward more negative value compared with the pristine materials (− 1.01 V). This behaviour is related to the formation of surface states at the interface of BiVO_4_–TiO_2_ heterojunction as it was also reported in other composite materials as ZrO_2_/TiO_2_, SnO_2_/TiO_2_ and WO_3_/BiVO_4_^61,62,63^. It has been observed that there is an increase in the photocurrent, along with the increase in the applied potential, and Hong et al.^59^ reported that this behaviour is unique for n-type semiconductors. Increase in the photocurrent increases the charge separation efficiency of the material^64^. In BiVO_4_/TiO_2_ nanocomposites (1:2.5 and 1:0.6), a displacement of E_fb_ toward lowest negative values was associated with the high BiVO_4_ content and the flat band alignment between BiVO_4_ and TiO_2_ (E_fb_ can provide information on the band positions of the semiconductor)^65^. Based on the values of flat band potentials and optical band gap energies, Fig. 10 shows the energy diagram proposed by coupling the BiVO_4_ and TiO_2_.

**Figure 11:**
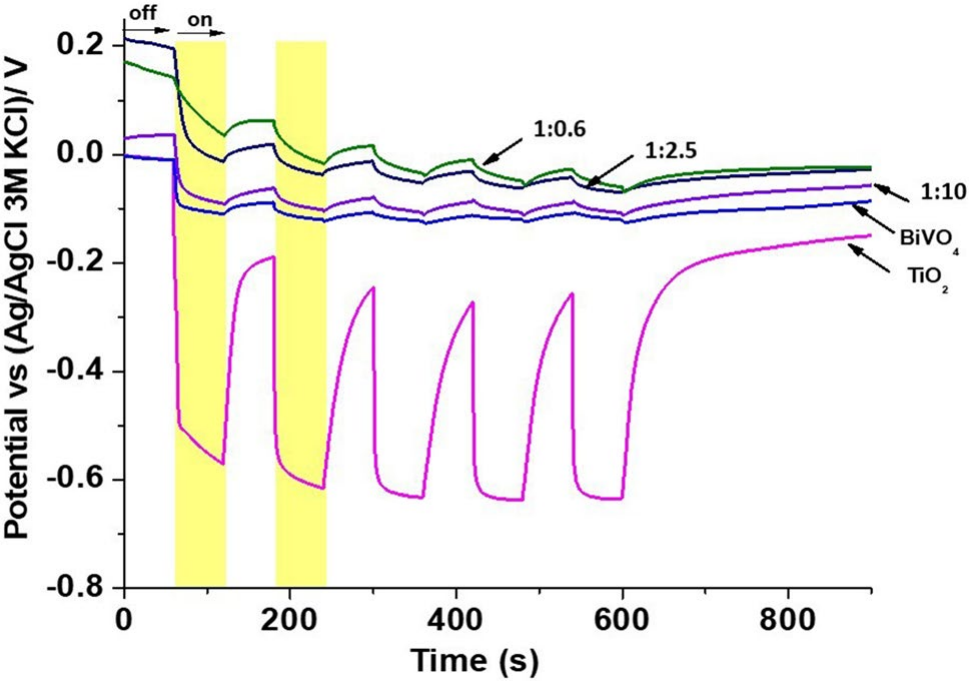
Time evolution of the open circuit potential (OCP) in the dark (off) and under UV illumination (on) for the synthetized nanocomposites.

Additionally, to evaluate the stability of the photo-generated carriers, open circuit potential (OCP) measurements under illumination of the synthetized materials were performed using visible light irradiation through 5 consecutive on–off cycles, previously bubbled with argon (Fig. 11). When the materials were irradiated, the OCP change toward more negative values confirms that the materials are *n*-type semiconductors which is supporting the observations from the Mott–Schottky plots; where observed an increased photocurrent with applied potential and also a further negative shift of the slopes. The results show that TiO_2_ have the highest negative value of OCP. This is associated with a major potential to reduce the photo-generated electrons that favors the formation of the superoxide and hydroxyl radicals required for the oxidation process. On the other hand, BiVO_4_ exhibits a lower negative value than TiO_2_ indicating that the electrons are accumulated in lower energy levels, these results are in agreement with the Mott Schottky measurements. The results of BiVO_4_/TiO_2_ nanocomposites show that the electrons are localized in lower energy levels than in the case of the pristine materials. Such levels are associated with the formation of surface states at the interface of BiVO_4_/TiO_2_ with a high BiVO_4_ content. In addition, when the illumination is interrupted, the OCP for BiVO_4_ and BiVO_4_/TiO_2_ nanocomposites recovers more slowly than TiO_2_ indicating that electrons are sluggishly transferred to species in the solution^66^.

**Figure 12:**
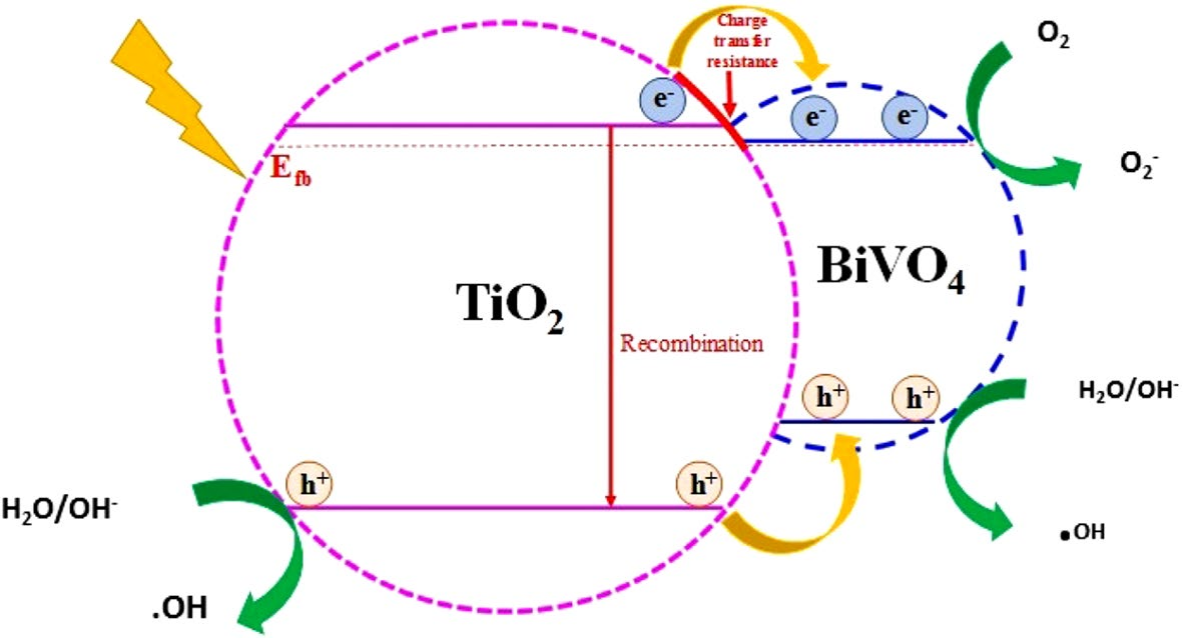
Scheme of BiVO_4_/TiO_2_ nanocomposite (1:10) and their interaction for spatial separation of photogenerated charge carriers.

According to the above results, the photo-generated electrons are transferred from TiO_2_ toward BiVO_4_ and the reverse for the photo-generated holes^67^. The photo-generated electrons in TiO_2_ have a major potential of reduction that can reduce surface chemisorbed O_2_ to yield strong oxidizing radicals (Fig. 12)^68^. However, it is important to mention that the material with low BiVO_4_ content exhibit the best behavior between the composites. This behavior is result of the flat band potential of the material and the potential reduction of its photogenerated electrons. Also, there observed an increase in the crystallite size of the nanocomposites with the increase in the concentration of the BiVO_4_ due to its comparatively higher atomic radius. Hence the composite with low concentration of BiVO_4_ (1:10) has shown a reduced crystallite size; resulted in more number of available crystallites per unit area and in addition to that, negative shift in the flat band potential which further provided more electrons for the reactions and improves the charge transfer properties of the material also contributed towards the good activity of the nanocomposites. The 1:10 ratio has shown a higher negative value for the flat band potential from other samples and also from the pristine materials this cause a significant difference in its photocatalytic efficiency. When the photons of sufficient energy incidents upon the catalyst surface, the electron hole pairs are created. Since the negative flatband potential provided a lower conduction band minima and hence the barrier is less for the charge separation and that favors more charge carriers over the surface of the material which will be available for the further redox reactions.

## Conclusion

Visible light photoactive BiVO_4_/TiO_2_ nanocomposites were synthesized using a modified sol–gel methodology which is a low temperature process that ensured the homogeneity, purity and stoichiometry of the products. The synthesized particles exhibited a homogeneous morphology with small crystallites compared to the parent materials (BiVO_4_ and TiO_2_) and good visible light response with reduced band gap, made the composites to absorb the major region of the sunlight spectra. The TEM images show regions with preferential orientations from all the characteristic planes of the composite. Moreover, two associated slab models resemble the alternating bright and dark signal channels from the monocilic (211) and tetragonal (004) plane stacking, generated during the grain growth, and this overlapped planes from both BiVO_4_ and TiO_2_ supporting the junction formation since the bright channels overlap in the same area marked with both reflections and. The effect of the incorporation of BiVO4 in the performance of the nanocomposite material under visible light was analyzed by synthesizing nanocomposites with the ratios BiVO_4_:TiO_2_ = 1:0.6, 1:2.5, and 1:10 and, it is observed that an increase in the efficiency of the composite with decreasing BiVO_4_ percentage. This behavior was associated to the low photocatalytic activity of BiVO_4_ due to the fast recombination of the photogenerated electron–hole pairs. The electrochemical Mott–Schottky analysis confirmed the formation of n-type semiconductors. The 1:10 nanocomposite have exhibited a comparatively higher negative value for the flat band potential (V_fb_ = −1.01 eV) which further results from a lower conduction band minimum which reduced the barrier for the charge separation and hence the photocatalytic efficiency of the composite by improving the charge transfer process. The above effects contribute to the higher performance of the composite, 1:10 with a quasi-complete removal of dyes with a concentration of 5 g/l for10 minutes of exposure under simulated visible light without the usage of any additional scavengers.
